# Morphological Characterization and SSR Profiling of Local Citrus Germplasm from Southern Croatia

**DOI:** 10.3390/plants15193008

**Published:** 2026-09-30

**Authors:** Ivana Paladin Soče, Mate Čarija, Rafael Montalt, Mara Marić, Katarina Hančević, Domagoj Ivan Žeravica, Mira Radunić

**Affiliations:** 1 Department for Mediterranean Plants, University of Dubrovnik, HR-20000 Dubrovnik, Croatia; mara.maric@unidu.hr (M.M.); domagoj-ivan.zeravica@unidu.hr (D.I.Ž.); 2 Institute for Adriatic Crops and Karst Reclamation, HR-21000 Split, Croatia; mate.carija@krs.hr (M.Č.); katarina.hancevic@krs.hr (K.H.); mira.radunic@krs.hr (M.R.); 3 Centro de Citricultura y Producción Vegetal, Instituto Valenciano de Investigaciones Agrarias (IVIA), 46000 Valencia, Spain; 4 Centre of Excellence for Biodiversity and Molecular Plant Breeding, Svetošimunska 25, 10000 Zagreb, Croatia

**Keywords:** citrus germplasm, Croatia, local accessions, morphological characterization, microsatellite markers

## Abstract

Local citrus germplasm is a valuable resource for biodiversity conservation and the preservation of traditionally maintained plant material. This study characterized 16 deliberately selected citrus accessions from private gardens in southern Croatia, each represented by a single tree, using 99 IPGRI descriptors and a panel of 15 SSR markers. The objectives were to identify the variables with the highest relative discriminating power, evaluate phenotypic relationships among the selected accessions, and determine their molecular correspondence with well-characterized reference cultivars. Phenotypic analyses identified 20 variables with the highest relative discriminating power. Hierarchical clustering and discriminant analysis of principal components (DAPC) revealed phenotypic structuring that broadly reflected the initial field-based horticultural group assignments. Multilocus SSR analysis identified five distinct 15-locus SSR profiles among the 16 Croatian accessions. The Croatian sweet orange, lemon, and sour orange accessions shared their respective 15-locus SSR profiles with the corresponding analyzed reference cultivars. Among the accessions initially assigned to the mandarin group, two distinct multilocus SSR profiles were identified: one was shared with the analyzed *Citrus clementina* reference cultivars, whereas the other was shared with the analyzed *Citrus deliciosa* reference cultivars. The observed variation should not be interpreted as an estimate of overall citrus diversity in southern Croatia because the accessions were selected in part for their morphological and pomological distinctiveness. The combined use of phenotypic and molecular characterization provides a basis for documentation, conservation, and further evaluation of local citrus germplasm.

## 1. Introduction

Local citrus germplasm represents a valuable genetic resource for biodiversity conservation and the preservation of traditionally maintained cultivars and accessions. Such germplasm may provide traits associated with tolerance to abiotic and biotic stresses and may contribute to the long-term sustainability of citrus production [1,2,3,4,5]. Accurate characterization and documentation are therefore essential for effective conservation and future utilization. However, the identification and classification of citrus germplasm remain challenging. The genus *Citrus* has an evolutionary history shaped by extensive interspecific hybridization, apomixis, a long history of cultivation, and widespread human-mediated dissemination. Consequently, genetic relationships within the genus are complex and only partially resolved [3,4,5,6,7]. In the present study, the sampled accessions were recorded under the local names traditionally used by growers, but their correspondence with well-characterized reference cultivars had not previously been established.

Historical records indicate that citrus cultivation in southern Croatia, particularly in the Dubrovnik area, dates back to the Middle Ages [8,9,10]. Its development was likely facilitated by the region’s proximity to Italy and its well-developed maritime trade routes [8,9,10]. Citrons (*Citrus medica* L.) were among the earliest citrus species cultivated on a larger scale in the Dubrovnik region. They were highly valued and sold individually at the local market. Archival records document court proceedings concerning the theft of individual fruits [11]. Renaissance travel writers and chroniclers reported the cultivation of citrons, lemons, and oranges within the enclosed gardens of patrician households in the Dubrovnik area [12,13,14]. However, plant-name inventories and botanical sources from the 19th and early 20th centuries generally referred to broader citrus types, such as citron, lemon, sweet lemon and sour orange, rather than to clearly defined cultivar names [15,16,17]. This historical pattern suggests that citrus germplasm in southern Croatia has long been maintained within traditional cultivation systems, frequently under local or generic names and without standardized pomological identification.

Today, traditional citrus trees in southern Croatia are still commonly maintained in private gardens and small orchards, often under local names, and many have not been systematically characterized. Previous classifications of citrus germplasm in Croatia were based predominantly on morphological traits and cultivation characteristics [18,19,20,21,22]. Although these studies provide valuable information on locally cultivated citrus material, they relied mainly on morphological traits and cultivation characteristics. The locally named accessions examined here had not previously been systematically compared with established citrus cultivars using standardized morphological descriptors and multilocus SSR markers. This uncertainty complicates the documentation, conservation, and management of Croatian citrus germplasm.

Standardized morphological descriptors enable the systematic assessment of vegetative, floral, fruit, and seed traits. They provide a common framework for comparing citrus germplasm, although environmental conditions and horticultural practices may influence the expression of some traits. SSR markers provide highly reproducible multilocus profiles that can be directly compared with reference material. They are therefore useful for citrus germplasm characterization and cultivar discrimination [23,24,25,26,27,28]. Together, morphological descriptors and SSR markers provide complementary information for characterizing traditionally maintained citrus germplasm. Accordingly, this study aimed to (i) identify the variables with the highest relative discriminating power, (ii) evaluate phenotypic relationships among the selected accessions, and (iii) determine their molecular correspondence with reference cultivars maintained in the IVIA citrus germplasm collection.

## 2. Results

### 2.1. Morphological Characterization

The complete morphological descriptor data and individual quantitative measurements for the 16 analyzed accessions are presented in Supplementary Table S1.

**Figure 1:**
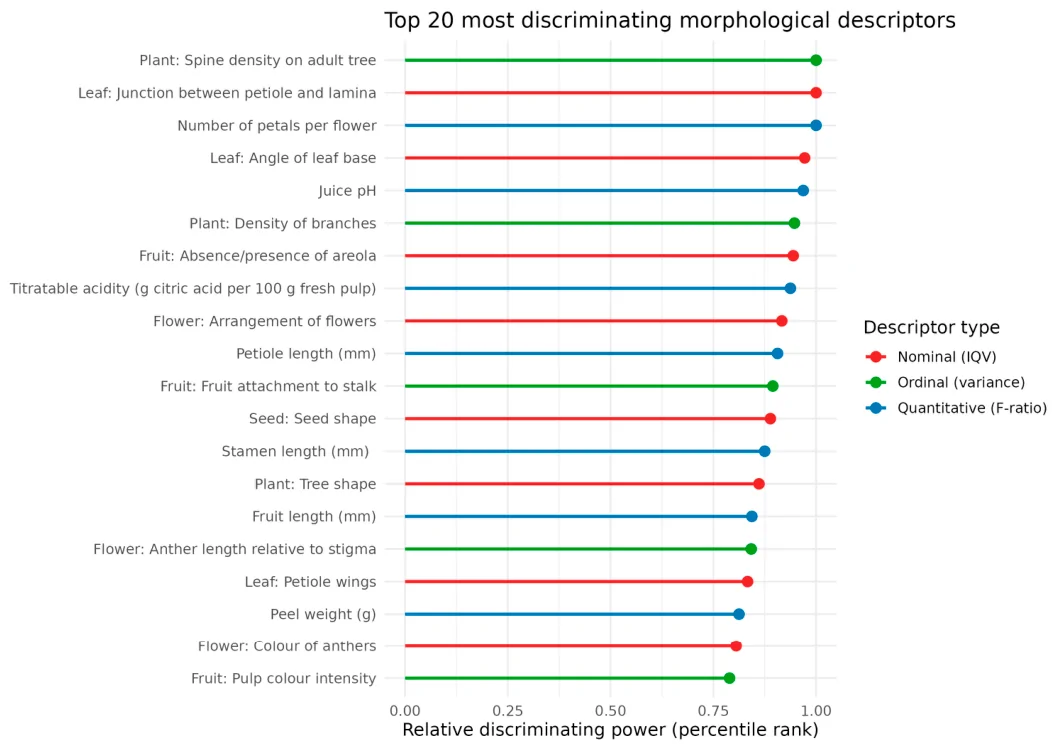
The 20 variables with the highest relative discriminating power among the analyzed citrus accessions, including morphological descriptors and supplementary physicochemical variables, ranked using a metric appropriate to each variable’s measurement scale (quantitative, ordinal, or nominal).

To identify the variables contributing most to phenotypic differentiation among the accessions, the evaluated morphological descriptors and supplementary physicochemical variables were ranked according to their relative discriminating power. The 20 highest-ranking variables are shown in Figure 1.

The variables differed in their relative ability to discriminate among accessions (Figure 1). Because they represented different measurement scales, quantitative variables were evaluated using the one-way ANOVA F-ratio, ordinal variables using variance, and nominal variables using the Index of Qualitative Variation (IQV). The scale-specific scores were converted to within-type percentile ranks, with lower rank values indicating higher scores within each variable type. The 20 variables with the lowest rank values were retained. Among the quantitative variables, number of petals per flower and juice pH ranked highest, while spine density on adult trees and branch density ranked highest among the ordinal variables. Junction between petiole and lamina and angle of leaf base ranked highest among the nominal variables.

**Figure 2:**
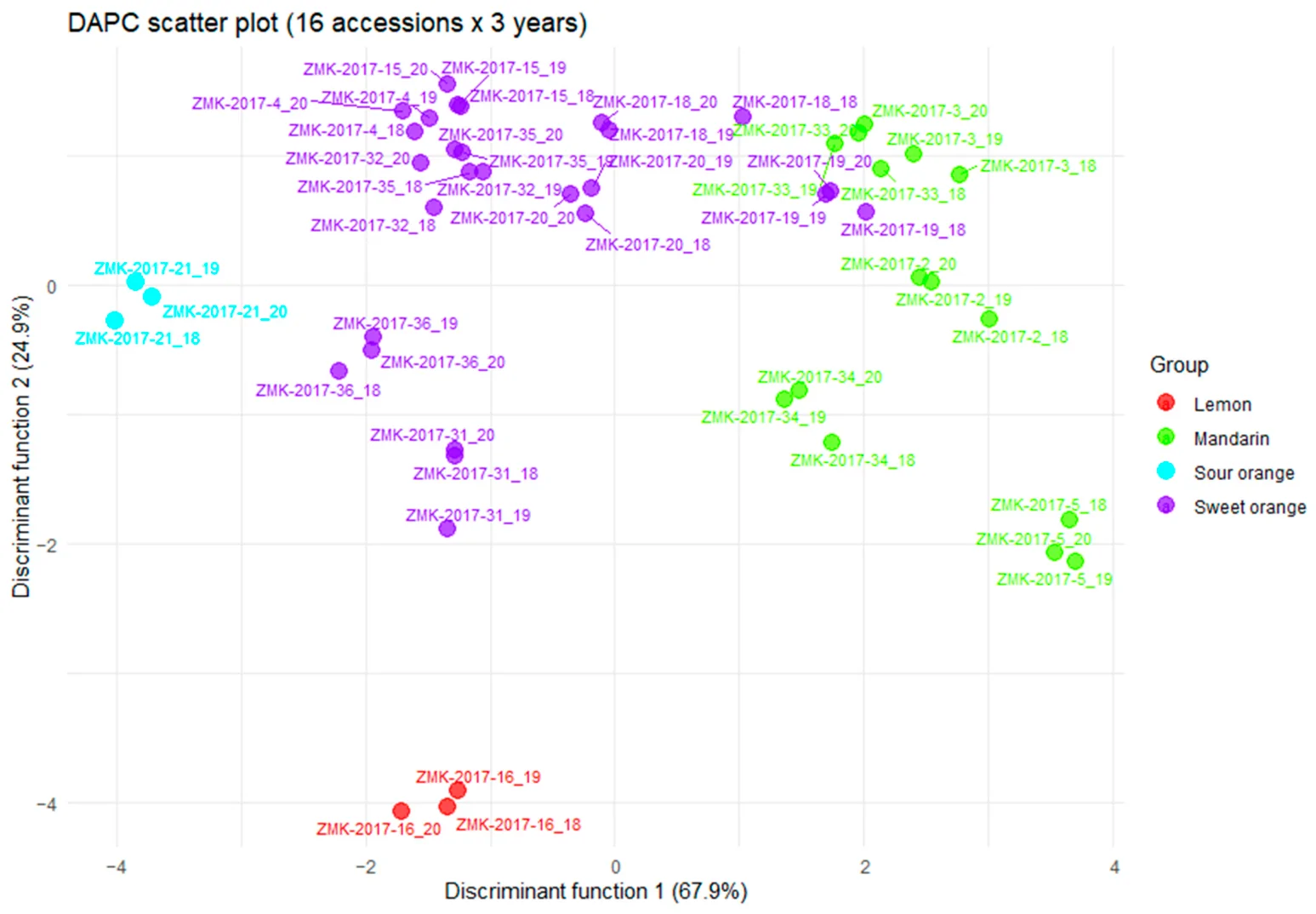
Scatter plot of local citrus accessions based on DAPC of the variables with the highest relative discriminating power (Gower dissimilarity, principal coordinate analysis, four principal coordinates retained, selected via a-score optimization). Each point represents one accession in one sampling year (2018–2020), colored by initial field-based horticultural group.

DAPC was performed using the 20 variables with the highest relative discriminating power. It included repeated annual observations of the same 16 trees from 2018 to 2020 and showed a pattern in discriminant space broadly consistent with the a priori field-based horticultural group assignments (Figure 2).

Accessions initially assigned to the Sweet orange group were located mainly in the central and negative region of the first discriminant axis, whereas mandarin accessions were positioned predominantly on its positive side. Both groups showed considerable within-group dispersion, particularly along the second discriminant axis, and partially overlapped. The single lemon and sour orange accessions occupied more distinct positions in the discriminant space. Because the Lemon and Sour orange groups were each represented by a single accession, their positions were interpreted descriptively and do not permit assessment of within-group variation. Overall, the DAPC pattern broadly reflected the initial field-based horticultural assignments, although some overlap among observations was evident. Year-specific DAPC analyses for 2018, 2019, and 2020 are presented in Supplementary Figure S1. They serve as exploratory comparisons of year-specific patterns, not as cross-validation or independent evidence of consistent group separation. For a limited number of categorical fruit- and seed-related descriptors, values observed in years with available material were retained for years when the material was unavailable. These values represent repeated accession-level information, not independent observations made in those years.

**Figure 3:**
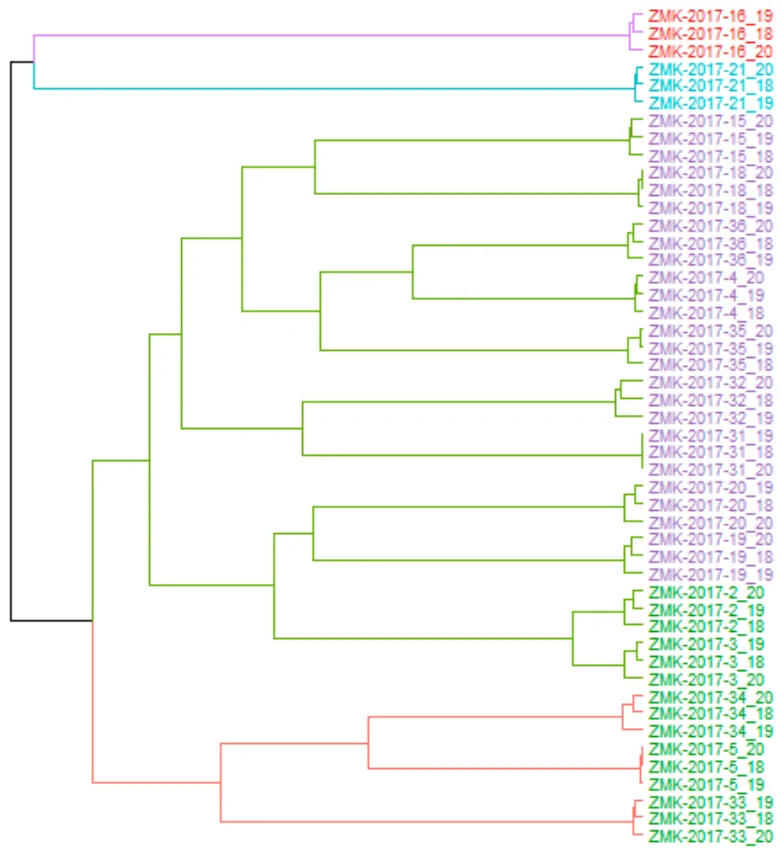
Hierarchical clustering of 48 accession × year observations (16 accessions observed over three years) based on the retained variables using Gower dissimilarity and average linkage.

Hierarchical clustering showed additional patterns of morphological similarity among the accession × year observations (Figure 3). ‘Mjesečar’ (ZMK-2017-16) and ‘Ljuta naranča’ (ZMK-2017-21) formed separate branches and joined the remaining observations at relatively high linkage levels. Most sweet orange observations were grouped within the main cluster. Within this cluster, ‘Ludica’ (ZMK-2017-15) grouped with one set of ‘Katarinka’ (ZMK-2017-18) observations, while ‘Ljetna’ (ZMK-2017-36) and ‘Trstenka’ (ZMK-2017-4) formed another closely related subgroup, with ‘Crvena’ (ZMK-2017-35) joining at a somewhat greater distance. A second set of ‘Katarinka’ (ZMK-2017-32) observations clustered with ‘Unknown name’ (ZMK-2017-31), whereas ‘Jaffa’ (ZMK-2017-20) and ‘Puh’ (ZMK-2017-19) formed neighboring subgroups. The ‘Lopudski aleksandrin’ observations (ZMK-2017-2, ZMK-2017-3, and ZMK-2017-33) were split between clusters: two sets occurred within the main cluster, while another grouped with the ‘Havana’ observations in a separate cluster. Accessions with the same local name did not always cluster together.

### 2.2. SSR Profiling

**Figure 4:**
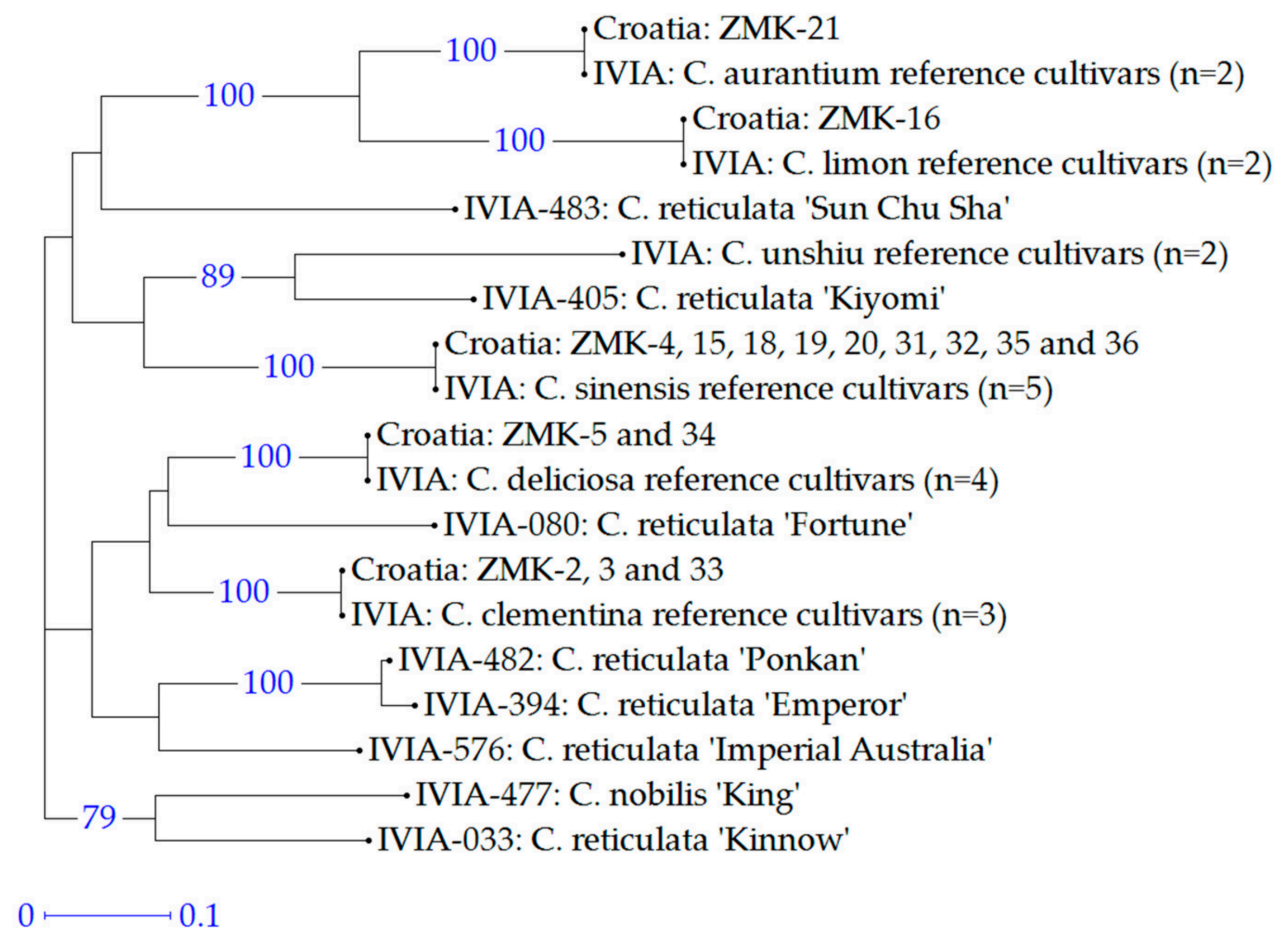
Weighted Neighbor-Joining dendrogram based on 15-locus SSR profiles of Croatian citrus accessions and reference cultivars. Allele sizes at the 15 SSR loci are provided in Supplementary Table S2b. Croatian accessions or IVIA reference cultivars displaying indistinguishable 15-locus SSR profiles within their respective sets were collapsed into single terminals. The dissimilarity matrix was calculated from allelic data using the Simple Matching index in DARwin 6.0.21. Numbers at nodes indicate bootstrap support values based on 1000 replicates. Bootstrap values ≥50% are shown. Bootstrap values refer to the stability of the groupings obtained by resampling SSR loci and should not be interpreted as evidence of cultivar identity. The scale bar represents Simple Matching dissimilarity. Collapsed IVIA reference terminals comprise the following cultivars: *C. aurantium* (n = 2), ‘Sevillano’ (IVIA-117) and ‘Afin Verna’ (IVIA-128); *C. limon* (n = 2), ‘Fino’ (IVIA-049) and ‘Eureka’ (IVIA-297); *C. unshiu* (n = 2), ‘Owari’ (IVIA-175) and ‘Okitsu’ (IVIA-195); *C. sinensis* (n = 5), ‘Navelina’ (IVIA-007), ‘Salustiana’ (IVIA-125), ‘Lane Late’ (IVIA-198), ‘Cara Cara’ (IVIA-300) and ‘Valencia Delta’ (IVIA-363); *C. clementina* (n = 3), ‘Hernandina’ (IVIA-012), ‘Clemenules’ (IVIA-022) and ‘Oronules’ (IVIA-132); and *C. deliciosa* (n = 4), ‘Mandarino Común’ (IVIA-154), ‘Tardivo di Ciaculli’ (IVIA-186), ‘Avana Apireno’ (IVIA-189) and ‘Salteñita’ (IVIA-361).

Multilocus SSR profiles were obtained for all Croatian accessions and reference cultivars. Allele sizes at the 15 SSR loci are presented in Supplementary Table S2. The resulting multilocus SSR profiles were used to construct the Weighted Neighbor-Joining dendrogram shown in Figure 4.

Across the 16 Croatian accessions, five distinct 15-locus SSR profiles were identified. The nine Croatian accessions initially assigned to the Sweet orange group (ZMK-2017-4, ZMK-2017-15, ZMK-2017-18, ZMK-2017-19, ZMK-2017-20, ZMK-2017-31, ZMK-2017-32, ZMK-2017-35 and ZMK-2017-36) exhibited identical multilocus SSR profiles across all 15 analyzed loci. The same 15-locus SSR profile was observed in the analyzed *C. sinensis* reference cultivars.

The Croatian accession initially assigned to the Sour orange group (ZMK-2017-21) shared the same 15-locus SSR profile as the analyzed *C. aurantium* reference cultivars. Likewise, the Croatian accession initially assigned to the Lemon group (ZMK-2017-16) shared the same 15-locus SSR profile as the analyzed *C. limon* reference cultivars.

Among the five Croatian accessions initially assigned to the Mandarin group (ZMK-2017-2, ZMK-2017-3, ZMK-2017-5, ZMK-2017-33 and ZMK-2017-34), two distinct multilocus SSR profiles were detected. Accessions ZMK-2017-2, ZMK-2017-3 and ZMK-2017-33 shared the same 15-locus SSR profile as the analyzed *C. clementina* reference cultivars, whereas accessions ZMK-2017-5 and ZMK-2017-34 shared the same 15-locus SSR profile as the analyzed *C. deliciosa* reference cultivars.

The Weighted Neighbor-Joining dendrogram based on 15-locus SSR profiles (Figure 4) provided a graphical representation of these molecular correspondences. Croatian accessions clustered together with reference cultivars sharing the same 15-locus SSR profiles. The groupings defined by these shared profiles received 100% bootstrap support, indicating strong support for the stability of these groupings. Additional relationships among the remaining mandarin-related reference cultivars received bootstrap support ranging from 79% to 100%. As these relationships were outside the scope of the present study, they were not considered further.

## 3. Discussion

This study documented phenotypic variation among the 16 deliberately selected citrus accessions. Multilocus SSR profiling identified five distinct molecular profiles among them. Phenotypic analyses based on standardized IPGRI descriptors [29] and supplementary physicochemical variables revealed differences among the selected accessions. SSR markers added information on their molecular correspondence with reference material. Morphological and molecular approaches are commonly combined in citrus germplasm characterization to assess phenotypic variation alongside multilocus molecular profiles [23,24,26,27,28,29,30,31].

### 3.1. Phenotypic Differentiation of Local Citrus Accessions

Phenotypic variation among the 16 selected accessions was evident across quantitative, ordinal, and nominal morphological descriptors, as well as the supplementary physicochemical variables included in the analysis. The 20 variables with the highest relative discriminating power encompassed vegetative, leaf, floral, fruit, seed, and physicochemical characteristics (Figure 2). Thus, the observed differentiation was not driven by a single group of traits. Similar variation in fruit- and seed-related morphological traits has also been reported in other citrus germplasm studies [2,32]. For example, Marboh et al. [33] reported substantial variation in seed and several fruit-related traits among 50 citrus genotypes. Endocarp morphology has also been used to characterize cultivars of other perennial fruit crops. Blazakis et al. [34] showed that olive endocarp morphology can be useful for cultivar identification because it is generally less affected by environmental conditions than fruit and leaf characteristics. However, the stability and discriminatory value of individual morphological traits cannot be established from the present dataset alone. The accessions were selected partly for their visible morphological and pomological distinctiveness, which may have contributed to the observed phenotypic variation.

The clustering pattern suggested that morphological similarity did not fully follow local naming or the initial horticultural assignments. The separation of ‘Mjesečar’ and ‘Ljuta naranča’ may reflect broader morphological differences among citrus groups. The positions of the ‘Lopudski aleksandrin’ observations indicate some phenotypic variability within the locally named material. These patterns should be interpreted as phenotypic similarities and differences rather than as evidence of genetic relatedness. Luro et al. [23] also highlighted the value of combining different characterization approaches in their study of the endemic Sardinian citrus Pompia. Several observations assigned to sweet orange occupied neighboring branches in the hierarchical clustering, although others were dispersed across the broader cluster. Sweet orange has been vegetatively propagated for centuries and is characterized by a relatively narrow genetic background associated with a common hybrid ancestry [35,36].

In the DAPC, the overall distribution broadly reflected the initial field-based horticultural assignments. Within-group dispersion was most apparent in the Sweet orange and Mandarin groups, which each contained multiple accessions. Because the Lemon and Sour orange groups each contained only one accession, within-group variation could not be assessed for those groups. These patterns should be interpreted as phenotypic structuring rather than as evidence of distinct biological or genetic groups. Variation among the sweet orange accessions is consistent with reports of differences in fruit characteristics despite the relatively narrow genetic background of cultivated sweet orange. Reported differences include fruit color, flavor, yield and maturity period [36], as well as fruit size and seed number [37]. For a limited number of fruit- and seed-related categorical descriptors, states observed in years with available material were retained for years without that material. Consequently, the multivariate patterns do not reflect independently observed values for every descriptor in every year.

The relatively distant position of ‘Ljuta naranča’ may partly reflect morphological characteristics associated with sour orange compared with the other citrus groups represented in the dataset. Pronounced phenotypic and chemical variation in *C. aurantium*, particularly in fruit-related traits, has also been reported [38]. The observed pomological variation requires cautious interpretation because fruit characteristics can be influenced by environmental conditions, crop load, canopy position, maturity stage, and harvest timing [36,39]. Ripening period is among the traits particularly sensitive to environmental conditions. The morphological differences observed here should therefore be regarded as phenotypic variation expressed under the conditions of the study area rather than as direct evidence of genetic differentiation [39].

Overall, because many morphological traits can be influenced by environmental and horticultural conditions, morphology alone cannot reliably establish genetic identity or relationships [23,40].

### 3.2. Molecular Characterization and SSR Profiling

The molecular uniformity observed among the Croatian sweet orange accessions is consistent with the well-documented difficulty of discriminating cultivated sweet oranges using molecular markers. Previous studies using DNA amplified fingerprinting [41], ISSR markers [42] and SSR markers [25,43] reported limited molecular differentiation among sweet orange cultivars. This pattern is consistent with genomic evidence indicating that cultivated sweet orange varieties share a narrow ancestral background and that much of their diversification has arisen through somatic mutations accumulated during long-term vegetative propagation [35,36]. Consequently, differences in local denomination or minor pomological characteristics do not necessarily correspond to differences in multilocus SSR profiles. In the present study, all nine Croatian accessions initially assigned to the Sweet orange group exhibited the same 15-locus SSR profile, which was also shared by the analyzed *C. sinensis* reference cultivars. This finding is consistent with the limited molecular differentiation reported for sweet orange. It also shows that SSR profiling can document traditionally maintained germplasm and its correspondence with reference material, even when closely related or clonally propagated accessions cannot be distinguished at the loci examined.

A similar interpretation applies to the Croatian sour orange and lemon accessions, which displayed 15-locus SSR profiles corresponding to those observed in the analyzed reference cultivars. Although the present study included only one Croatian accession representing each of these horticultural groups, the results demonstrate the usefulness of multilocus SSR profiling for relating locally maintained germplasm to well-characterized reference material. In the case of lemon, the observed molecular correspondence is consistent with previous reports of limited molecular polymorphism within cultivated lemon groups [3]. Molecular characterization was particularly informative for the accessions initially assigned to the Mandarin group. Multilocus SSR profiling resolved two distinct molecular profiles: one was shared with the analyzed *C. clementina* reference cultivars, whereas the other was shared with the analyzed *C. deliciosa* reference cultivars. Within each of these groups, the Croatian accessions exhibited identical profiles at all 15 SSR loci examined, consistent with the limited molecular differentiation previously reported for clonally propagated citrus groups including clementines [25]. These findings therefore do not indicate high molecular diversity within the Croatian accessions initially assigned to the Mandarin group. Rather, they show how SSR profiling complements morphological characterization by revealing molecular correspondences that field observations and local names alone cannot reliably establish.

An additional consideration is the complex evolutionary history of cultivated citrus. Genomic studies show that many cultivated citrus types arose through hybridization and introgression, with mandarins showing particularly complex admixture patterns [35]. This provides useful context for the two SSR profiles detected among accessions initially assigned to the Mandarin group, corresponding to *C. clementina* and *C. deliciosa* reference material. However, these molecular correspondences should not be interpreted as evidence of ancestry, as the 15-SSR panel was used to assess molecular correspondence rather than to reconstruct evolutionary history. The Weighted Neighbor-Joining dendrogram provided a visual summary of the molecular correspondences identified through multilocus SSR profiling. The clustering of Croatian accessions with reference cultivars sharing the same 15-locus SSR profiles, together with the high bootstrap support values, provided strong support for the stability of the recovered groupings. The remaining relationships among the reference cultivars were not considered further because the objective of the present study was to characterize Croatian citrus germplasm rather than to infer phylogenetic or evolutionary relationships within the genus *Citrus*.

The present results also illustrate both the strengths and limitations of SSR markers for citrus germplasm characterization. Multilocus SSR profiles provide highly reproducible molecular data that are useful for documenting correspondences among accessions and reference cultivars. However, identical 15-locus SSR profiles should not be interpreted as evidence of complete genetic identity, because somatic mutations affecting agronomically important traits may occur without altering the microsatellite loci analyzed. Accordingly, in the present study, the term “molecular correspondence” specifically refers to an identical allelic profile at the 15 SSR loci examined in a Croatian accession and a reference cultivar. When several reference cultivars shared an identical 15-locus SSR profile, the observed molecular correspondence did not allow cultivar-specific identification of the Croatian accessions. Likewise, the absence of polymorphism at the analyzed loci does not preclude phenotypic variation among clonally propagated cultivars, as previously reported for clonally propagated citrus groups, including sweet oranges and clementines [35,36].

Consequently, multilocus SSR profiling is most informative when interpreted together with complementary sources of evidence, particularly standardized morphological characterization. The integration of these approaches provides a more reliable basis for documenting traditionally maintained citrus germplasm and for identifying accessions that warrant further evaluation and conservation.

### 3.3. Conservation Implications

The results highlight the importance of documenting traditionally maintained citrus accessions from private gardens and small orchards in southern Croatia. These accessions are relevant for conservation because of their local history, traditional use, and observed phenotypic variation. Similar studies of Mediterranean citrus germplasm have also reported substantial phenotypic, chemical, and genetic variation in traditional material [2,23,32,44].

## 4. Materials and Methods

### 4.1. Plant Material and Location

Sixteen local citrus accessions were selected from private gardens and traditional cultivation sites in southern Croatia (Table 1). Selection was based on their long-term maintenance, recognition by local growers, presumed affiliation with different citrus horticultural groups, and observed morphological and pomological distinctiveness. For most of the analyzed accessions, detailed morphological and molecular characterization had not previously been performed, and their precise geographical origin could not be confirmed. Therefore, the accessions were considered locally maintained citrus germplasm. Their preliminary horticultural group assignments were based on field observations and information provided by the growers. The prefix “ZMK” refers to the internal accession coding system established during the initial field survey. The codes identify individual trees maintained in situ at their original locations on private properties rather than in an ex situ collection. Sampling was conducted with the verbal permission of the respective tree owners.

**Figure 5:**
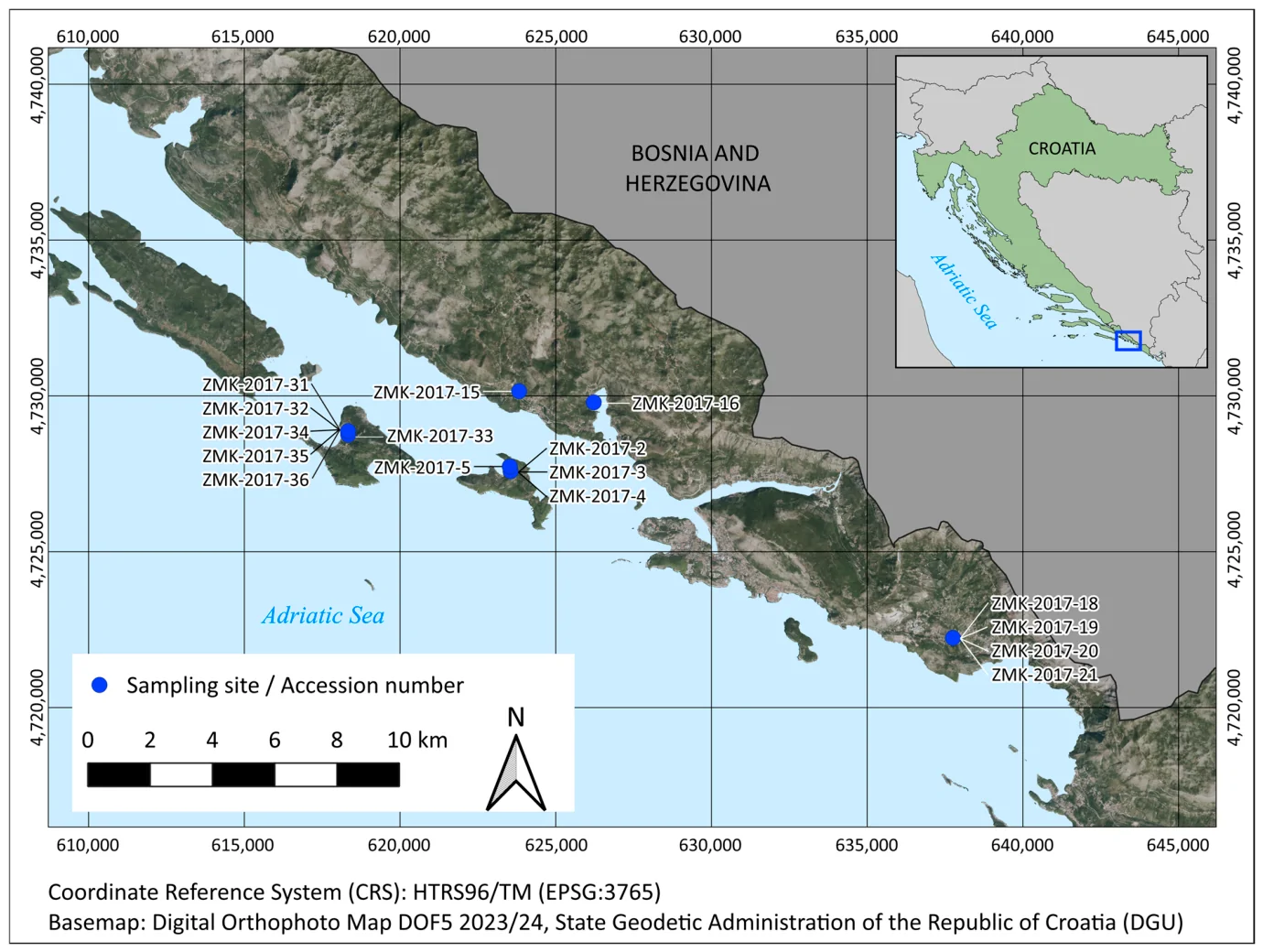
Sampling locations in Dubrovnik-Neretva County.

The analyzed trees were estimated to be at least 50 years old. They were grown under low-input management, outside intensive commercial orchards and without a standardized production system. They generally had no irrigation systems, and pruning and other management practices were infrequent and irregular. Detailed information on crop load, soil characteristics, and other site-specific management conditions was not systematically recorded. Information on rootstock use was obtained from the tree owners, based on their knowledge of the planting and grafting history. According to the owners, the older trees were grafted onto sour orange rootstock. The study was conducted from 2018 to 2020 in Dubrovnik-Neretva County, southern Croatia (Figure 5).

### 4.2. Morphological Characterization and Fruit Sampling

Morphological characterization was performed using 99 citrus traits according to the IPGRI descriptor list [29], comprising 31 quantitative, 21 ordinal, and 47 nominal descriptors. These covered plant, leaf, flower, fruit, and seed characteristics. The accession-level morphological descriptor states are provided in Supplementary Table S1a, the original quantitative measurements in Supplementary Tables S1b,e, and the year-specific nominal and ordinal descriptor states in Supplementary Table S1i. Summary statistics for quantitative morphological and physicochemical traits are provided in Supplementary Table S1f. Total soluble solids (TSSs), titratable acidity (TA), pH, sugar content, and peel weight were also measured in the sampled fruits. These supplementary physicochemical variables were not among the 99 IPGRI descriptors. They were evaluated for discriminating power and could be selected for multivariate analyses.

Each accession was represented by a single tree, the biological unit of the study (n = 16). The accession × year records are repeated annual observations of these trees, not independent biological replicates. The same trees were followed throughout the 2018–2020 study period. Plant- and shoot-related descriptors were assessed on the same individual tree representing each accession. For leaf and flower characterization, 10 individual leaves and 10 individual flowers were examined per accession in the sampling years when the corresponding material was available. Seed characterization was based on individual seeds obtained from the sampled fruits. The number of seeds examined varied among accessions and years according to seed production. Fruits, leaves, and flowers collected from each tree were treated as within-tree subsamples, not as independent biological replicates. Nominal and ordinal descriptors were assessed independently for each accession and sampling year whenever the corresponding material was available. When a descriptor was assessed in all three years, identical states represent repeated observations of that state rather than values copied from one year. For a limited number of categorical fruit- and seed-related descriptors in the final multivariate dataset, the accession-level state observed in another year was retained when the material needed for assessment was unavailable. The retained values are repeated accession-level information, not observations made independently in that year. In the year-specific descriptor table (Supplementary Table S1i), years without the corresponding material are indicated as ND.

The aim was to sample 20 fruits per accession each year, from throughout the canopy, to obtain a representative sample for assessing fruit morphological traits. Of these, 10 fruits per accession and year were used for the detailed quantitative fruit measurements reported in Supplementary Table S1b. The corresponding qualitative fruit descriptor states are provided in Supplementary Tables S1a,i. Given the age and variable condition of the trees, field surveys indicated that 20 fruits per accession and year was a feasible target whenever sufficient fruit was available. Because the investigated accessions differed in their ripening periods, fruits were not collected on a common calendar date. When sampling was possible, each accession was sampled during its customary harvest period, as reported by its owner. Available sampling dates for each accession and year are provided in Supplementary Table S1g. Fully developed fruits representative of the typical harvest stage of each accession were selected for analysis, corresponding to BBCH phenophase 89. Following sampling, maturity was further characterized using total soluble solids (TSSs), titratable acidity (TA), and juice yield. The available maturity-related measurements for each accession × year combination are also reported in Supplementary Table S1g. These measurements documented the maturity of the sampled fruits and were not used in advance to determine sampling dates. The measurements were interpreted with reference to the citrus maturity criteria applicable during the study period [45]. Annual fruit sampling could not be completed for all accession × year combinations because of field-related limitations. The trees of accessions ZMK-2017-5 and ZMK-2017-31 were severely damaged by extreme winds and storm conditions during the winter of 2018/2019, preventing subsequent fruit sampling. In addition, ZMK-2017-18 produced an insufficient number of fruits in 2018 to meet the target sample size. No quantitative fruit or juice measurements were available for ZMK-2017-5 and ZMK-2017-31 in 2019 and 2020 or for ZMK-2017-18 in 2018. These unavailable quantitative observations were recorded as missing values and were not replaced by zeros or numerically imputed.

#### 4.2.1. Tree and Leaf Characteristics

Quantitative tree and canopy descriptors included trunk/rootstock diameter ratio and spine length. Qualitative descriptors comprised tree growth habit, canopy density, branch characteristics, shoot tip color, and thorn morphology. Leaf measurements included lamina length and width, lamina length-to-width ratio, petiole length, and petiole wing width. Measurements were obtained using the WinFOLIA image analysis system (Regent Instruments Inc., Quebec, QC, Canada), a computer-based system designed for quantitative analysis of leaf morphology.

Qualitative descriptors included leaf shape, margin type, apex shape, color intensity, petiole wing characteristics, and vein prominence.

#### 4.2.2. Flower Characteristics

Flower characterization included quantitative measurements of pedicel length, calyx diameter, petal dimensions, number of petals and stamens, and flower buds per inflorescence. Floral dimensions were measured using a digital caliper (PRO+ 1930, BGS Technic, Wermelskirchen, Germany) with an accuracy of 0.02 mm, while petal measurements were obtained using the WinFOLIA Pro 2019a image analysis software (Regent Instruments Inc., Quebec, QC, Canada).

Qualitative floral descriptors included flowering time, flower type, flower color, inflorescence type, and filament and style characteristics.

#### 4.2.3. Fruits and Juice Characteristics

Quantitative fruit descriptors included fruit weight and dimensions, rind thickness, areola diameter, juice content, number of segments, seed number, and seed morphometric characteristics. Fruit and peel weights were determined using an analytical balance (ABS 220-4, KERN & Sohn GmbH, Balingen, Germany; accuracy ±0.01 g). Fruit dimensions and peel thickness were measured using a digital caliper. After the peel was separated, the number of segments per fruit was determined manually.

Qualitative fruit descriptors included fruit shape, peel color and texture, albedo adherence, pulp color, fruit apex and base shape, seed characteristics, and bearing habit.

Juice was obtained by manually pressing each individual fruit, and juice volume was recorded in milliliters. Juice yield was calculated as the volume of juice obtained per unit fruit mass and expressed as mL 100 g^−1^ fresh fruit weight: juice yield = [juice volume (mL)/fruit weight (g)] × 100. Juice quality was assessed based on total soluble solids (TSSs), titratable acidity (TA), and pH. Total soluble solids were determined using a digital refractometer (OPTi Digital Brix 85, Bellingham + Stanley, Tunbridge Wells, UK) at 20 °C and expressed as °Brix.

Titratable acidity (TA) was determined using 12.5 g of extracted fruit pulp. The sample was mixed with 50 mL of boiled distilled water and filtered through filter paper. A 10 mL aliquot of the filtrate was titrated with 0.1 M NaOH. As the pH approached 7.0, NaOH was added more slowly until an endpoint of pH 8.1 ± 0.2 was reached. Each determination was performed in duplicate, and the mean of the two measurements was used for further analysis. Total acidity was expressed as grams of citric acid per 100 g of fresh pulp (g citric acid 100 g^−1^ fresh pulp). Sugar content was determined using an enzymatic method [46]. Glucose, fructose, and sucrose were quantified using a commercially available enzyme kit (K-SUFRG 04/18, Megazyme Ltd., Bray, Co. Wicklow, Ireland). Absorbance was measured using a UV-1600PC spectrophotometer (VWR, Radnor, PA, USA), and sugar concentrations were expressed in g L^−1^. pH was measured using a pH meter (SevenEasy S20, Mettler Toledo, Columbus, OH, USA).

### 4.3. DNA Extraction and SSR Analysis

Genomic DNA was extracted from young leaves using the PureLink™ Plant Total DNA Purification Kit (Invitrogen™, Carlsbad, CA, USA) according to the manufacturer’s instructions. DNA quality and concentration were assessed spectrophotometrically and verified by agarose gel electrophoresis.

The molecular analysis comprised 16 Croatian citrus accessions and 26 well-characterized reference cultivars maintained in the citrus germplasm collection of the Instituto Valenciano de Investigaciones Agrarias (IVIA, Valencia, Spain). All samples were genotyped using a panel of 15 informative simple sequence repeat (SSR) markers. The reference cultivars were selected to represent the citrus horticultural groups corresponding to the initial field assignments of the Croatian accessions. A broader set of mandarin and mandarin-related reference cultivars was included because of the complex genetic background of this group. The complete list of reference cultivars and their multilocus SSR allele sizes is provided in Supplementary Table S2b.

To ensure direct comparability of allele sizes, all Croatian accessions and reference cultivars were amplified in the same laboratory using identical PCR conditions, capillary electrophoresis settings, internal size standards, and allele-scoring procedures. Information on the SSR loci, including linkage group assignment, primer sequences, GenBank accession numbers, sequence lengths, annealing temperatures and original references, is provided in Supplementary Table S2a.

Polymerase chain reactions (PCRs) were performed using a Mastercycler ep gradient S thermocycler (Eppendorf, Hamburg, Germany) in a final reaction volume of 15 μL containing 0.5 U of Taq DNA polymerase (Fermentas, Waltham, MA, USA), 30 ng of genomic DNA, 0.2 μM of each forward (fluorescently labeled) and reverse primer, 0.2 mM of each dNTP, 1× PCR reaction buffer, and 1.5 mM MgCl_2_. PCR amplification consisted of an initial denaturation at 94 °C for 5 min, followed by 40 cycles of denaturation at 94 °C for 30 s, primer annealing at either 50 or 55 °C for 30 s depending on the marker, and extension at 72 °C for 30 s, with a final extension at 72 °C for 8 min.

Amplified fragments were separated by capillary electrophoresis using a CEQ™ 8000 Genetic Analysis System (Beckman Coulter Inc., Brea, CA, USA). PCR products were denatured at 90 °C for 2 min before injection at 2 kV for 30 s, followed by fragment separation at 6 kV for 35 min. Allele sizes were determined using a 400 bp internal size standard and analyzed with GenomeLab™ version 10.0 software (Beckman Coulter Inc., Brea, CA, USA). Electropherograms were visually inspected prior to allele assignment. Whenever amplification failed or allele scoring remained uncertain after review, the complete SSR genotyping was repeated with appropriate controls. Final allele calls were manually reviewed before establishing the multilocus SSR profiles.

SSR allele sizes were compiled into a diploid allelic matrix containing data for all Croatian accessions and reference cultivars. Each SSR locus was represented by the two allele values of the diploid genotype, with allele order treated as interchangeable. The final 15-locus dataset contained no missing allele calls. No treatment of missing data was required. Genotypes were analyzed directly as diploid allele values without conversion to allele presence/absence coding.

Pairwise dissimilarities were calculated from these allelic data using the Simple Matching index in DARwin version 6.0.21 [47,48]. A Weighted Neighbor-Joining dendrogram was constructed from the resulting dissimilarity matrix to provide a distance-based graphical representation of multilocus SSR relationships among Croatian accessions and reference cultivars. Bootstrap support was estimated from 1,000 replicates generated by resampling SSR loci with replacement, and bootstrap values ≥ 50% were displayed.

### 4.4. Statistical Analysis

Descriptive statistics for quantitative morphological traits were calculated using Microsoft Excel for Microsoft 365 and R software version 4.3.1 (R Core Team, Vienna, Austria, 2024). The variable set evaluated for discriminating power comprised the 99 IPGRI morphological descriptors together with the supplementary quantitative fruit and juice physicochemical characteristics. For quantitative descriptors based on multiple within-tree measurements, individual fruit, leaf, flower, and seed measurements were first summarized at the accession × year level. The ANOVA F-ratios were calculated from these annual tree-level values, not from individual organ measurements. To identify the variables contributing most to accession differentiation, discriminating power was assessed using a metric appropriate to each variable’s measurement scale: a one-way ANOVA F-ratio (between-accession relative to within-accession year-to-year variance) for quantitative descriptors, variance for ordinal descriptors, and the Index of Qualitative Variation (IQV) for nominal descriptors.

Descriptors showing no observed variation or with insufficient usable data for calculation of the corresponding scale-specific statistic were excluded prior to percentile ranking. Scores were converted to within-type percentile ranks, with lower rank values indicating higher scale-specific scores. The 20 variables with the lowest rank values were retained for multivariate analyses. In Figure 1, higher plotted values indicate greater relative discriminating power. The ANOVA F-ratio, ordinal variance, and IQV quantify different properties and were not interpreted as statistically equivalent measures. Percentile transformation was used only as a pragmatic ranking procedure to place the scale-specific statistics on a common relative ranking scale for variable selection. The original scale-specific score and corresponding percentile rank for all variables included in the variable-ranking analysis are provided in Supplementary Table S1j.

The same final set of 20 retained variables was used consistently for Gower dissimilarity, PCoA, hierarchical clustering, DAPC and the separate annual exploratory analyses. The final 48 accession × year × 20-variable analytical matrix is provided in Supplementary Table S1h, and the corresponding Gower dissimilarity matrix is provided in Supplementary Table S1k. Gower dissimilarity was calculated using pairwise deletion of missing values. For each pair of accession × year records, only descriptors with non-missing data in both records contributed to the distance. No numerical imputation was applied to missing quantitative observations. Hierarchical clustering was performed on the Gower dissimilarity matrix using average linkage.

Discriminant Analysis of Principal Components (DAPC) was performed using the R package adegenet 2.1.11 [49] and the a priori field-based horticultural group assignments reported in Table 1. The Gower dissimilarity matrix based on the retained 20-variable dataset was subjected to principal coordinate analysis (PCoA), and the resulting principal coordinates were used as numeric input for DAPC. A-score optimization selected four principal coordinates for the final DAPC. All available discriminant functions were retained (n = 3 for the four a priori horticultural groups). For the longitudinal multivariate analyses, annual accession × year observations from 2018–2020 were retained separately, yielding 48 records (16 accessions × 3 years). These 48 records represent repeated annual observations of the same 16 trees and were not treated as 48 independent biological replicates. DAPC was additionally performed separately for each sampling year as an exploratory comparison of year-specific patterns (Supplementary Figure S1). These annual analyses were not used as cross-validation or as independent evidence of consistent group separation.

## 5. Conclusions

Morphological analyses revealed phenotypic variation among the accessions, while SSR profiling identified five distinct molecular profiles and revealed correspondence between the Croatian material and reference cultivars representing sweet orange, lemon, sour orange, clementine, and common mandarin. Because the accessions were deliberately selected and each was represented by a single tree, the observed variation should not be considered representative of overall citrus diversity in southern Croatia. Likewise, shared SSR profiles should not be interpreted as complete genetic identity. The combined use of phenotypic and molecular characterization provides a useful basis for documenting and conserving locally maintained citrus germplasm, while future studies using broader sampling and higher-resolution genomic approaches may further resolve variation among closely related accessions.

## Figures and Tables

**Table 1:** Local citrus accessions included in the study, with accession code, local name, and initial field assignment based on field observations and information provided by local growers.

Accession Code	Local Name	Initial Field Assignment
ZMK-2017-2	Lopudski aleksandrin	Mandarin
ZMK-2017-3	Lopudski aleksandrin	Mandarin
ZMK-2017-4	Trstenka	Sweet orange
ZMK-2017-5	Havana	Mandarin
ZMK-2017-15	Ludica	Sweet orange
ZMK-2017-16	Mjesečar	Lemon
ZMK-2017-18	Katarinka	Sweet orange
ZMK-2017-19	Puh	Sweet orange
ZMK-2017-20	Jaffa	Sweet orange
ZMK-2017-21	Ljuta naranča	Sour orange
ZMK-2017-31	Unknown name	Sweet orange
ZMK-2017-32	Katarinka	Sweet orange
ZMK-2017-33	Lopudski aleksandrin	Mandarin
ZMK-2017-34	Havana	Mandarin
ZMK-2017-35	Crvena	Sweet orange
ZMK-2017-36	Ljetna	Sweet orange

## Data Availability

The data supporting the findings of this study are provided in the article and its Supplementary Materials. Further inquiries can be directed to the corresponding author.

## Supplementary Materials

The following supporting information can be downloaded at https://www.mdpi.com/article/10.3390/plants15193008/s1, Table S1a–k: Morphological descriptor data, original quantitative measurements, summary statistics, sampling dates, year-specific descriptor states, variable-ranking statistics, final analytical matrix, and Gower dissimilarity matrix; Figure S1, Year-specific DAPC scatter plots for 2018, 2019, and 2020; Table S2a,b: SSR markers used for genotyping and allele sizes (bp) obtained at 15 SSR loci in Croatian citrus accessions and reference cultivars.
