# Supplementary material for: Morphological Characterization and SSR Profiling of Local Citrus Germplasm from Southern Croatia
Source: Plants (Basel). 2026 Sep 30;15(19):3008. doi: 10.3390/plants15193008 (PMC13666969; doi:10.3390/plants15193008)
Supplement: Supplementary file 1 [file plants-15-03008-s001.zip › Supplementary files 28_9/Figure S1.pdf]

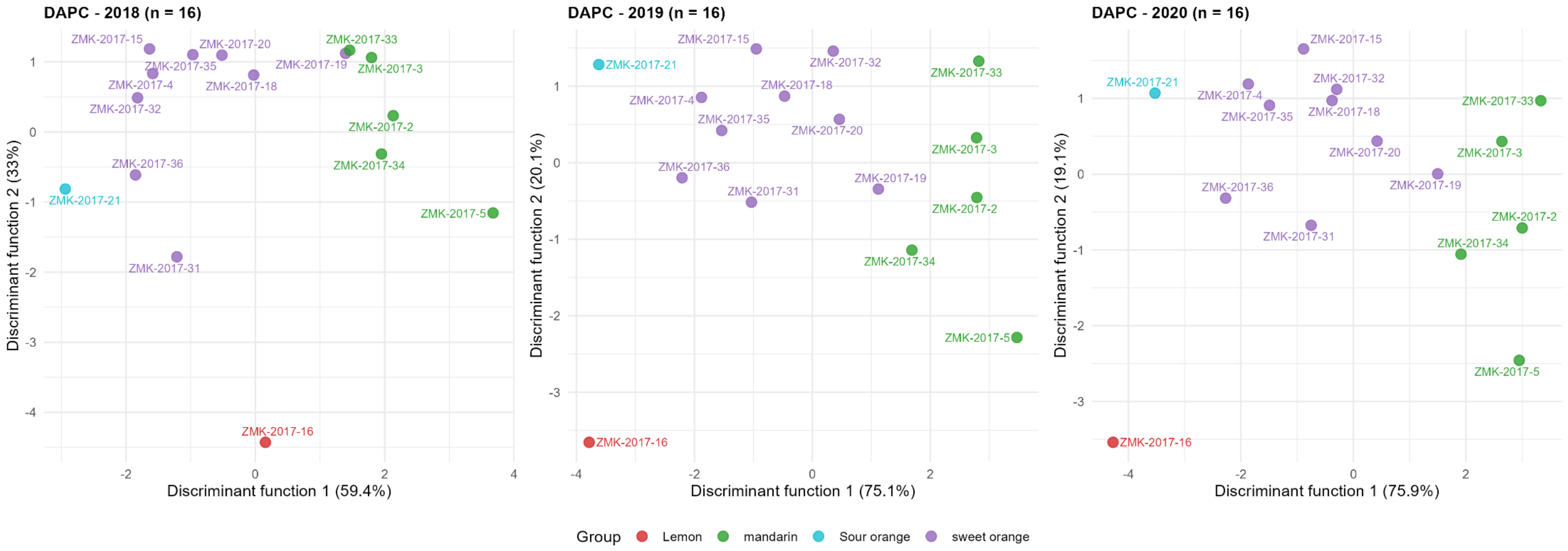

Figure S1: Year-specific DAPC scatter plots for 2018, 2019 and 2020 based on the final set of 20 retained variables. Each panel represents an exploratory analysis of the 16 accessions for the respective sampling year. These analyses are presented as exploratory comparisons of year-specific patterns and should not be interpreted as cross-validation or as evidence of consistent group separation.
